# Vanishing Bile Duct Syndrome as a Rare Complication of Classic Hodgkin's Lymphoma: A Case Report and Literature Review

**DOI:** 10.7759/cureus.100708

**Published:** 2026-01-03

**Authors:** Jody W Tai, Vivek D Shah, Rhett L Harmon, Shaun Chandna, Jessica Yan, Payman Fathizadeh, Simon Beaven, Deepthi Karunasiri, Phyllis Kim

**Affiliations:** 1 Internal Medicine, Olive View University of California Los Angeles Medical Center, Los Angeles, USA; 2 Hematology and Oncology, Olive View University of California Los Angeles Medical Center, Los Angeles, USA; 3 Gastroenterology, Olive View University of California Los Angeles Medical Center, Los Angeles, USA; 4 Pathology, Olive View University of California Los Angeles Medical Center, Los Angeles, USA

**Keywords:** abvd chemotherapy, acute liver failure (alf), classic hodgkin lymphoma, paraneoplastic phenomenon, vanishing bile duct syndrome

## Abstract

Vanishing bile duct syndrome (VBDS) is an acquired condition of progressive intrahepatic bile duct loss, resulting in severe cholestasis. Here, we present a case of VBDS in a patient with newly diagnosed classic Hodgkin's lymphoma (cHL). Our patient presented with cholestatic liver test elevations, including bilirubin and alkaline phosphatase. Liver biopsy confirmed the paucity of bile ducts and did not show involvement by lymphoma. Though the exact pathophysiology is unclear, it is postulated to be driven by cytokine release and T-lymphocyte cell dysregulation. Our patient has made a remarkable, albeit incomplete, liver recovery with cHL-directed treatment. Given the poor prognosis and higher mortality rate of VBDS, a multidisciplinary approach involving oncology, hepatology, and prompt initiation of dose-adjusted chemotherapy is imperative.

## Introduction

Vanishing bile duct syndrome (VBDS) is a syndrome of progressive destruction and loss of intrahepatic bile ducts leading to cholestasis. Presentations can range from asymptomatic to fulminant liver failure. Most commonly, VBDS arises within one to six months after a drug-induced liver injury, most commonly with antibiotics such as amoxicillin/clavulanic acid, fluoroquinolones, or trimethoprim-sulfamethoxazole, among others [1]. In order to diagnose VBDS, alternate etiologies of biliary duct loss, such as primary biliary cholangitis, primary sclerosing cholangitis, and graft-versus-host disease, must be ruled out. The diagnosis is confirmed with a liver biopsy, which shows intralobular bile ductopenia with less than 50% of portal areas having a bile duct in an adequate biopsy specimen of at least 10 portal tracts [1]. A paucity of reports has described VBDS associated with classic Hodgkin's lymphoma (cHL), with no consensus in the literature review on its incidence. The pathobiology is poorly understood but likely to be driven by hepatotoxic cytokines and thymocyte-lymphocyte (T-lymphocyte) dysregulation, leading to immunogenic damage to biliary ducts. Due to impaired liver function due to VBDS, treatment of the underlying disease is often challenging. Dose modifications can lead to undertreatment of the cHL, which is a highly treatable and curable disease. Standard of care with chemotherapy and radiation has resulted in high cure rates, with a greater than 80% five-year overall survival [2]. In our patient, treatment adaptations have led to remarkable improvements in liver function in parallel with the response of the lymphoma. We add to the existing literature a case of cHL as the leading explanation for VBDS with ensuing cholestatic liver injury and provide a structured literature review to inform diagnostic and therapeutic decisions.

## Case presentation

A 31-year-old male with no prior medical history presented to the emergency department for diffuse abdominal pain and diarrhea for one week. He reported one week of subjective fevers and three to four watery bowel movements daily. He also endorsed 20-pound weight loss and occasional night sweats over the prior six months and left-sided neck swelling over the prior four months. He denied any emesis, early satiety, dysphagia, odynophagia, melena, hematochezia, or dark-colored urine. There was no reported family history of liver disease. Social history was notable for working in construction and no tobacco, alcohol, or drug use.

Upon presentation, the patient’s temperature was 36.7 degrees Celsius, heart rate of 80 beats per minute, blood pressure of 112/66 millimeters of mercury (mmHg), and oxygen saturation of 99% on room air. Physical exam was significant for left-sided neck fullness without any palpable lymphadenopathy elsewhere. The patient was not noted to be jaundiced.

He was found to have leukopenia, thrombocytopenia, elevated liver tests, and coagulopathy. Laboratory investigations are summarized in Table 1. Other relevant studies included a stool polymerase chain reaction (PCR) panel for infectious causes of diarrhea, a stool ova and parasite exam, and a stool acid-fast bacillus (AFB) exam. Liver injury workup included negative anti-nuclear antibody, anti-smooth muscle antibody, hepatitis panel, alpha-1 antitrypsin, and normal iron stores. At presentation, the R factor was 1.8, suggestive of a cholestatic pattern of injury.

**Table 1:** Laboratory results on admission WBC: white blood cell, Hgb: hemoglobin, LDH: lactate dehydrogenase, INR: international normalized ratio, AST: aspartate aminotransferase, ALT: alanine aminotransferase, GGT: gamma-glutamyl transferase

Test	Result	Reference Range
WBC	4.3 K/cumm	4.5-10.0 K/cumm
Hgb	12.8 g/dL	12.0-14.6 g/dL
Platelets	64 K/cumm	160-360 K/cumm
Alkaline phosphatase	398 U/L	38-126 U/L
AST	68 U/L	15-41 U/L
ALT	234 U/L	14-54 U/L
Total bilirubin	6.4 mg/dL	0.1-1.2 mg/dL
Direct bilirubin	4.2 mg/dL	0.1-0.4 mg/dL
GGT	90 U/L	7-50 U/L
HIV	Negative	Negative
Hepatitis B	Negative	Negative
Hepatitis C	Negative	Negative
Anti-mitochondrial antibody	Negative	Negative
Anti-smooth muscle antibody	Negative	Negative

**Figure 1:**
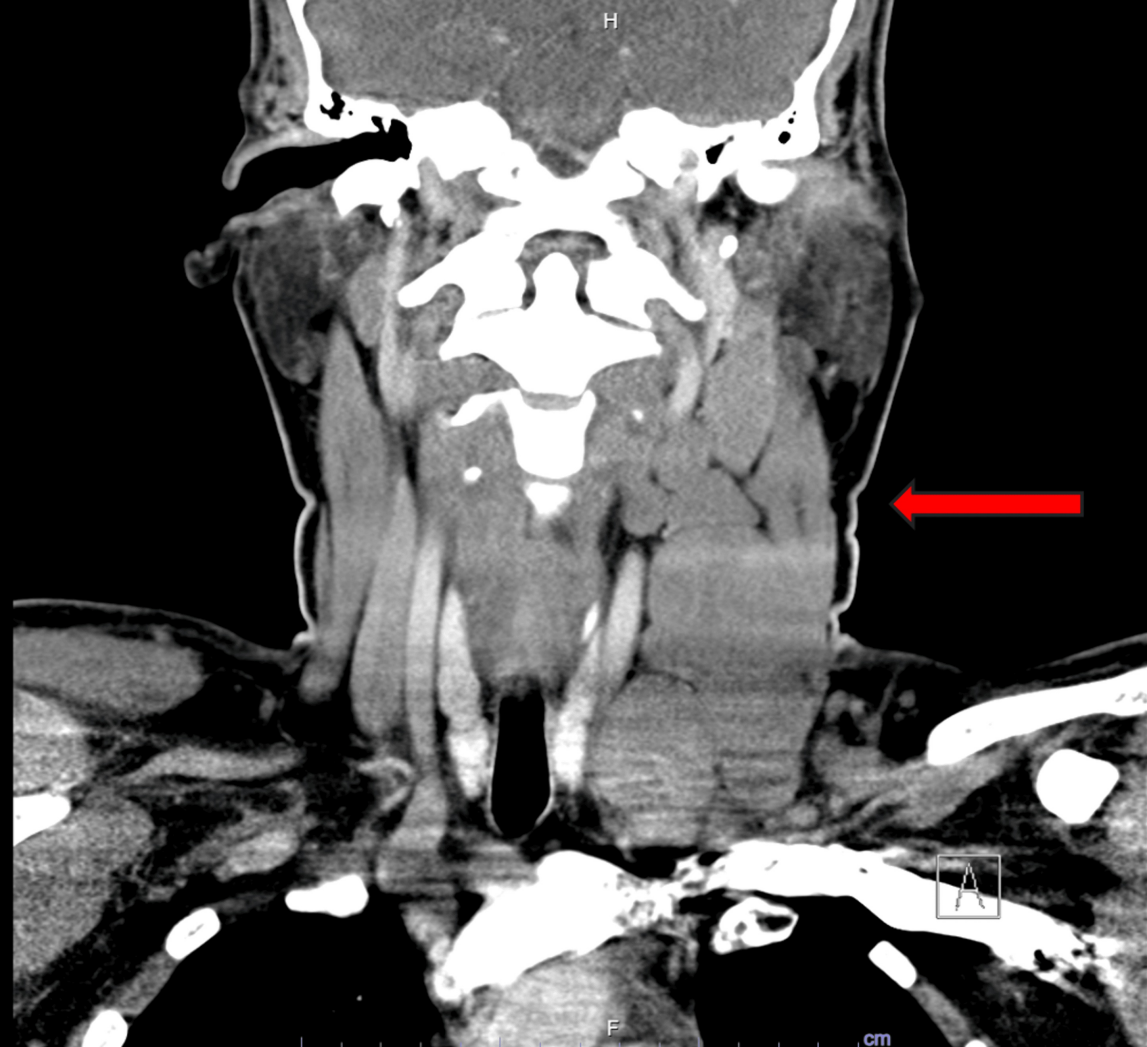
Coronal view of CT neck The red arrow highlights the left cervical lymphadenopathy.

Computerized tomography (CT) of the neck was significant for enlarged, bulky cervical lymph nodes throughout the left, largest in the left neck measuring 5.2 x 2.7 x 4.1 centimeters (cm), as shown in Figure 1. 

**Figure 2:**
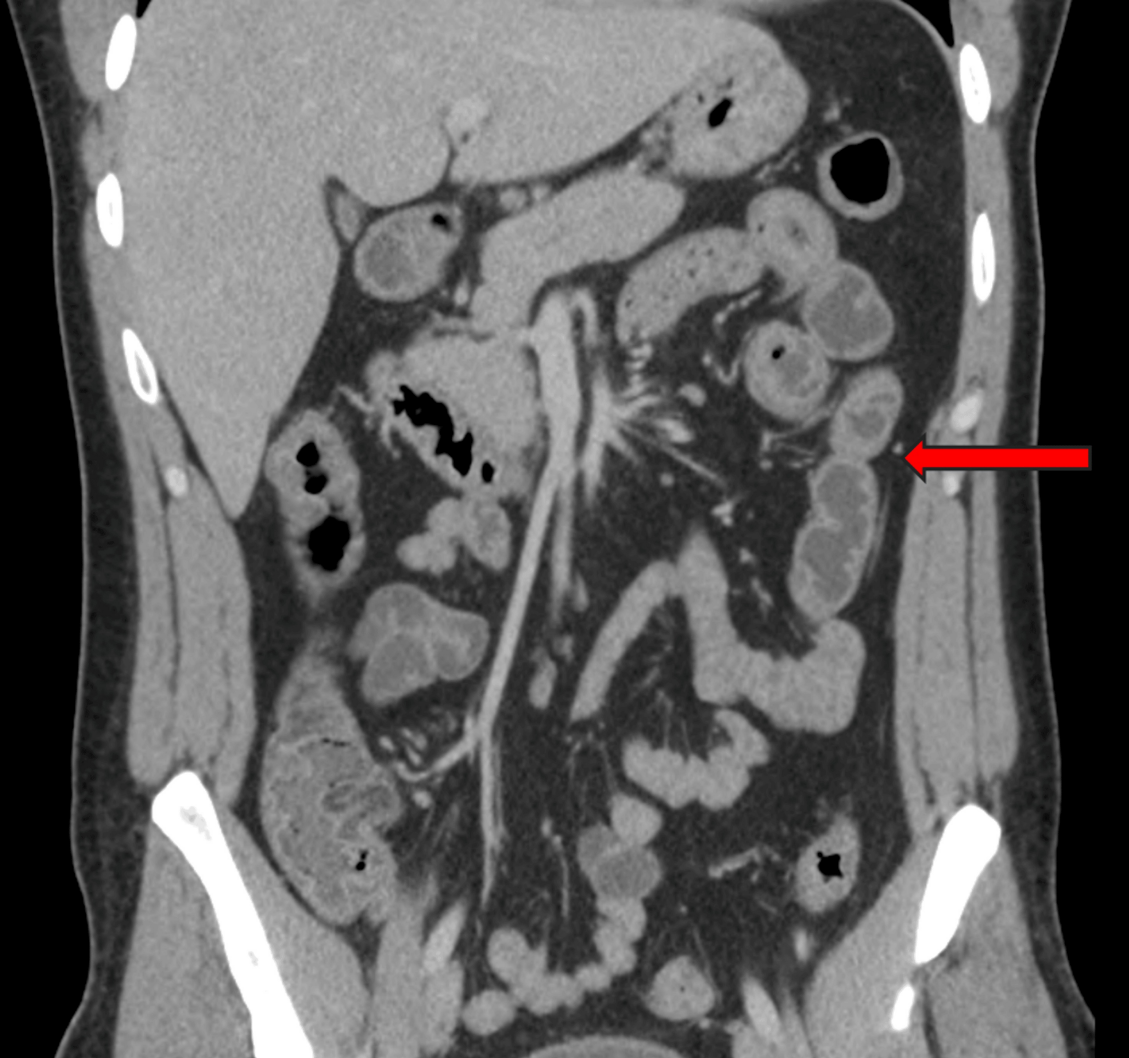
CT scan of the chest, abdomen, and pelvis with inflammatory changes in a section of the jejunum, as well as in the terminal ileum and colon The red arrow points towards an area of mucosal enhancement with increased wall thickness.

CT scan of the chest, abdomen, and pelvis revealed inflammatory changes in a section of the jejunum, as well as in the terminal ileum and colon, as seen in Figure 2.

**Figure 3:**
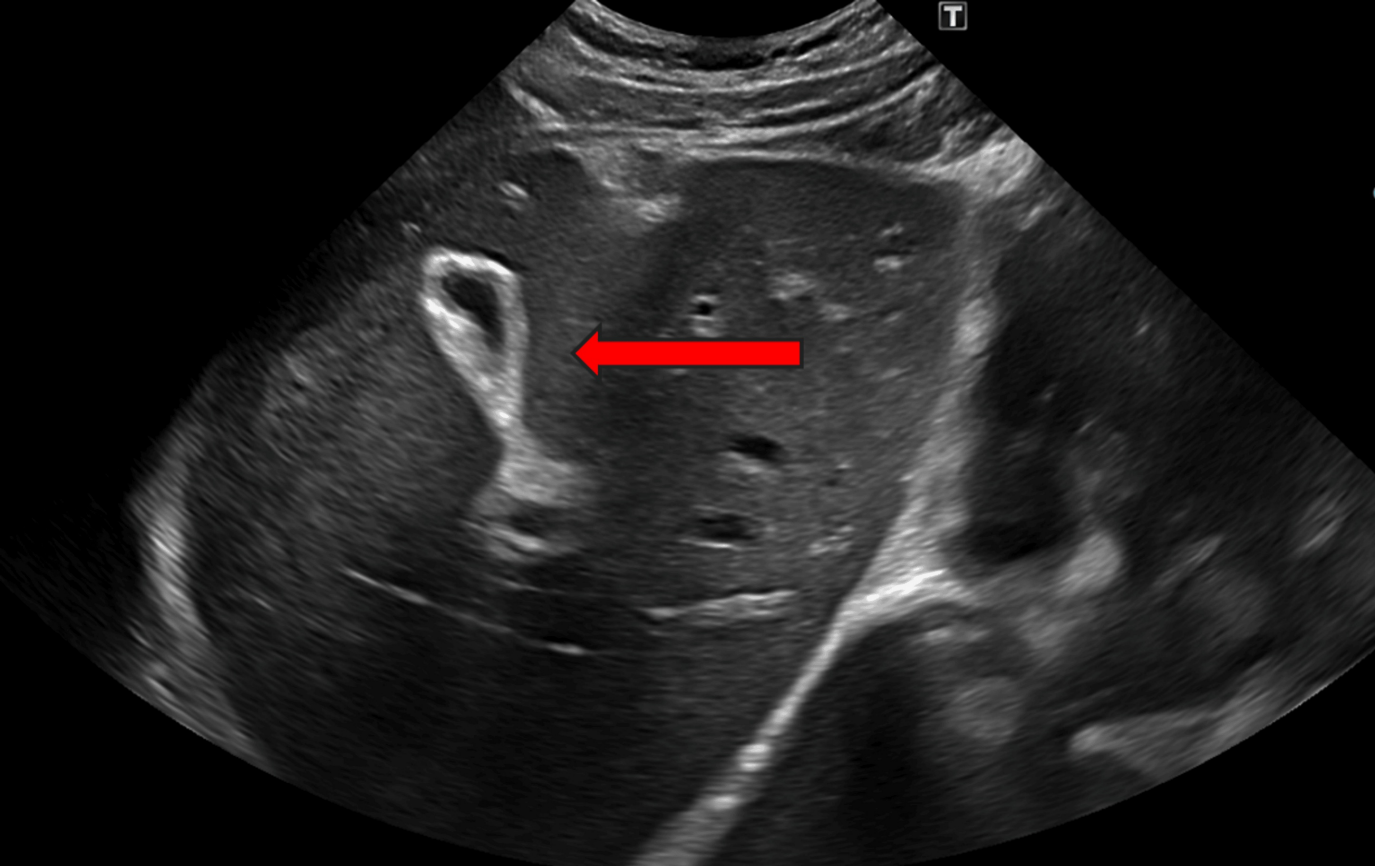
Ultrasound of the liver from the long axis showing hepatomegaly The red arrow points towards contracted gallbladder.

The right upper quadrant ultrasound was consistent with hepatomegaly and a contracted gallbladder, as seen in Figure 3.

**Figure 4:**
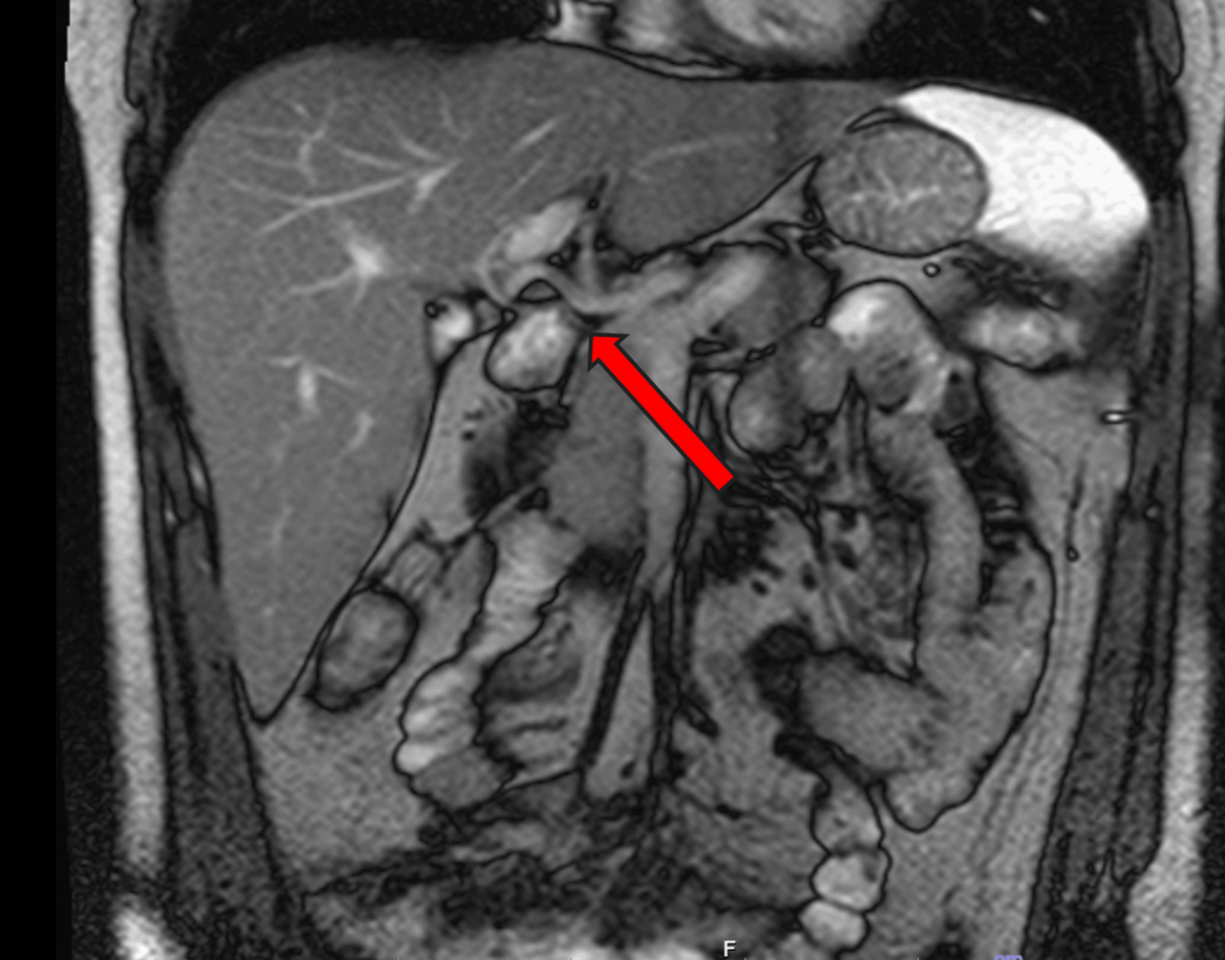
MRCP showing normal bile ducts and a red arrow pointing towards a patent biliary tree without evidence of strictures, masses, or biliary filling defects MRCP: magnetic resonance cholangiopancreatography

Magnetic resonance cholangiopancreatography (MRCP) demonstrated normal bile ducts and a patent biliary tree without evidence of strictures, masses, or biliary filling defects; there was no evidence of primary sclerosing cholangitis, as seen in Figure 4. 

Gastroenterology was consulted and performed an endoscopy with colonoscopy that revealed patchy colonic inflammation with ulcerations and erythema in the ascending and transverse colon, with biopsies showing active inflammation without granulomas or lymphomatous involvement. Enteroscopy demonstrated mild gastropathy and diffuse erythema, edema, and ulcerations throughout the duodenum and jejunum, which were biopsied to evaluate for *Helicobacter pylori *and rule out Crohn’s disease. Biopsies and staining were negative for cytomegalovirus (CMV) but positive for *H. pylori*, for which the patient was initiated on quadruple therapy involving tetracycline, metronidazole, bismuth salicylate, and pantoprazole. A biopsy of the left-sided neck mass revealed lymphoid tissue with Reed-Sternberg cells and positive CD15 and CD30 markers, confirming classic Hodgkin’s lymphoma of the nodular sclerosis type. A bone marrow biopsy was negative for malignant involvement.

**Figure 5:**
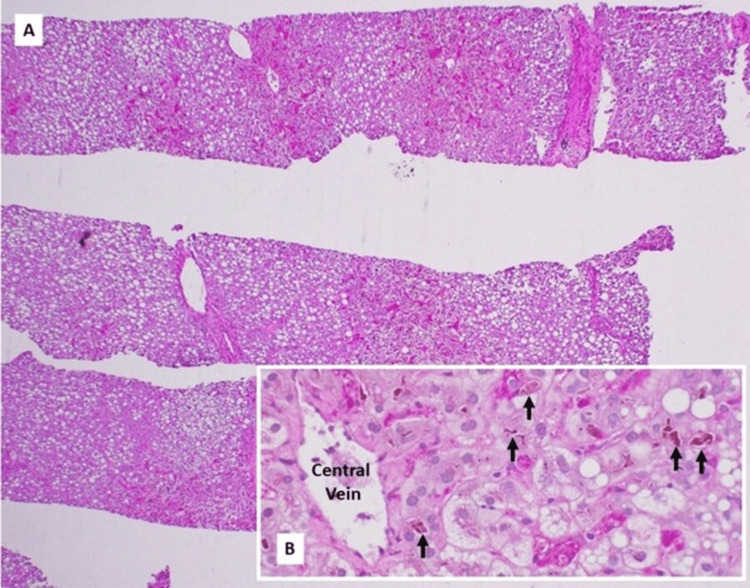
Liver core biopsies demonstrating macrovesicular steatosis (A, periodic acid-Schiff stain with diastase, 40×) and marked canalicular-predominant cholestasis (B, arrow; periodic acid-Schiff stain with diastase, 400×) Credit: Payman Fathizadeh, MD

**Figure 6:**
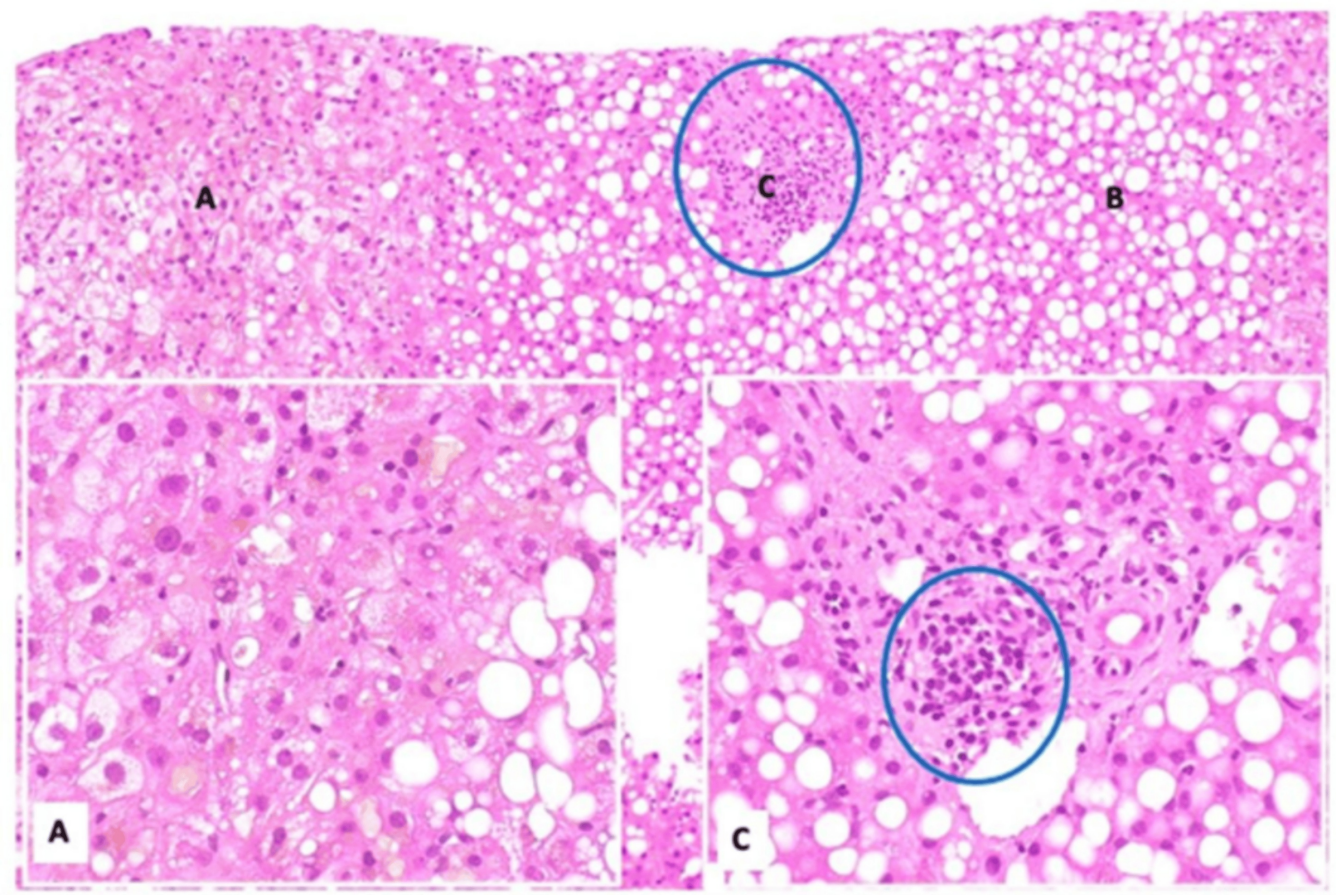
Liver core biopsies demonstrate canalicular cholestasis (A, hematoxylin and eosin stain, 400x), approximately 70% macrovesicular steatosis (B, hematoxylin and eosin stain, 100x), and significant bile duct loss (C, hematoxylin and eosin stain, 400x) The few remaining bile ducts exhibit evidence of injury and degenerative changes. These histologic findings are consistent with ductopenia/vanishing bile duct syndrome, which may be associated with drug-induced liver injury or the patient's documented history of Hodgkin lymphoma. The concurrent macrovesicular steatosis may also represent a manifestation of drug-related hepatotoxicity. No evidence of lymphomatous infiltration is identified. The histologic features do not suggest extrahepatic biliary obstruction. Credit: Payman Fathizadeh, MD

The patient continued to decline with worsening cholestatic liver injury and physical signs of hepatic dysfunction, including jaundiced skin and sclera, despite empiric treatment with ursodiol. No other causes of liver injury, such as viral hepatitis, autoimmune hepatitis, primary biliary cholangitis, primary sclerosing cholangitis, or inherited conditions, were found on workup, including serologies and imaging. A liver biopsy was pursued, which showed bile duct injury accompanied by ductopenia affecting more than 50% of the portal tracts and severe macrovesicular steatosis without fibrosis, as seen in Figures 5-6. No histologic evidence of direct lymphomatous invasion was found.

The patient denied any use of herbal remedies, over-the-counter drugs, or other culprit medication use that implicated concern for drug-induced liver injury. Given the absence of other identifiable etiologies, the findings of cholestatic liver injury and severe ductopenia on liver biopsy were thought to most likely represent VBDS as a paraneoplastic phenomenon in the setting of newly diagnosed Hodgkin's lymphoma (HL). At this juncture, given extremely poor liver function and dependence on hepatic metabolism for the use of most active agents in the treatment of cHL, we had an extensive discussion with the patient and his family. We shared that not treating the disease would likely result in fulminant liver failure. However, treatment of the disease with optimal therapy would likely result in severe treatment-related toxicities, amplifying known cytopenias, emetogenesis, cardiotoxicities, and more. The option of hospice was introduced to the patient, given worsening liver function tests, and he opted to continue with full care, given that he is the sole provider for his two children.

**Figure 7:**
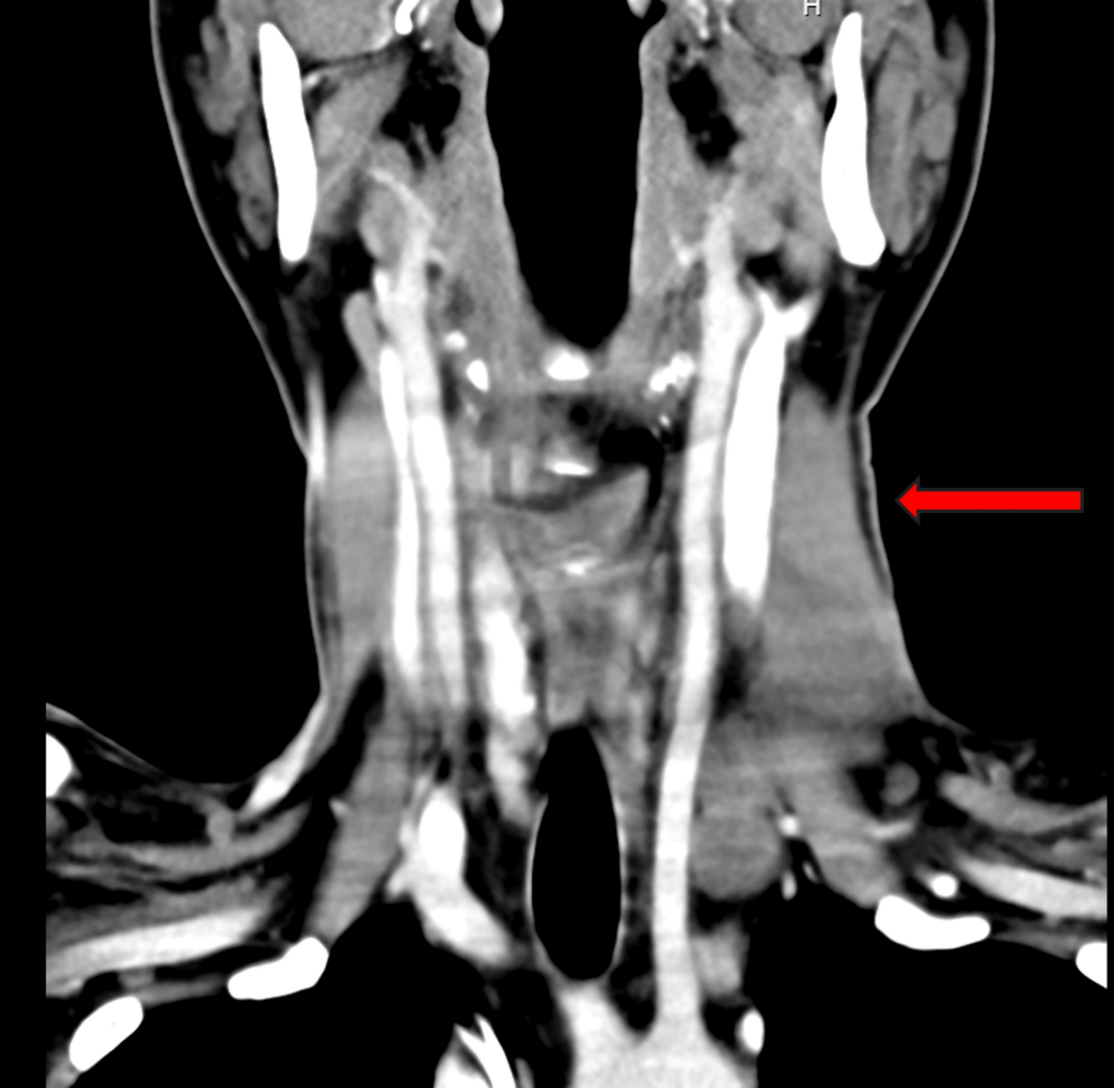
Interval imaging of the CT neck showing marked decrease in the size and number of left jugulodigastric chain adenopathy as labeled by the red arrow, largest measuring 13 mm x 24 mm x 38 mm

The patient was initiated on chemotherapy with cyclophosphamide, brentuximab vedotin, and dexamethasone. He received one cycle of inpatient and was discharged with ursodiol. Liver function improved with one dose of therapy, and treatment was accordingly continued with additional cHL-specific treatments, including brentuximab vedotin, vinblastine, and dacarbazine. These drugs were chosen due to their known efficacy and ability to modify the doses in the setting of impaired liver function. Doxorubicin and bleomycin were omitted due to the risk of severe toxicities, which could further augment mortality risk. At the time of writing this article, our patient has completed four cycles of chemotherapy with a peak in liver function tests around cycle 2 and subsequent downtrending with additional cycles of chemotherapy. Interval imaging with CT of his neck showed a marked decrease in the size of his left neck lymphadenopathy, as seen in Figure 7.

**Figure 8:**
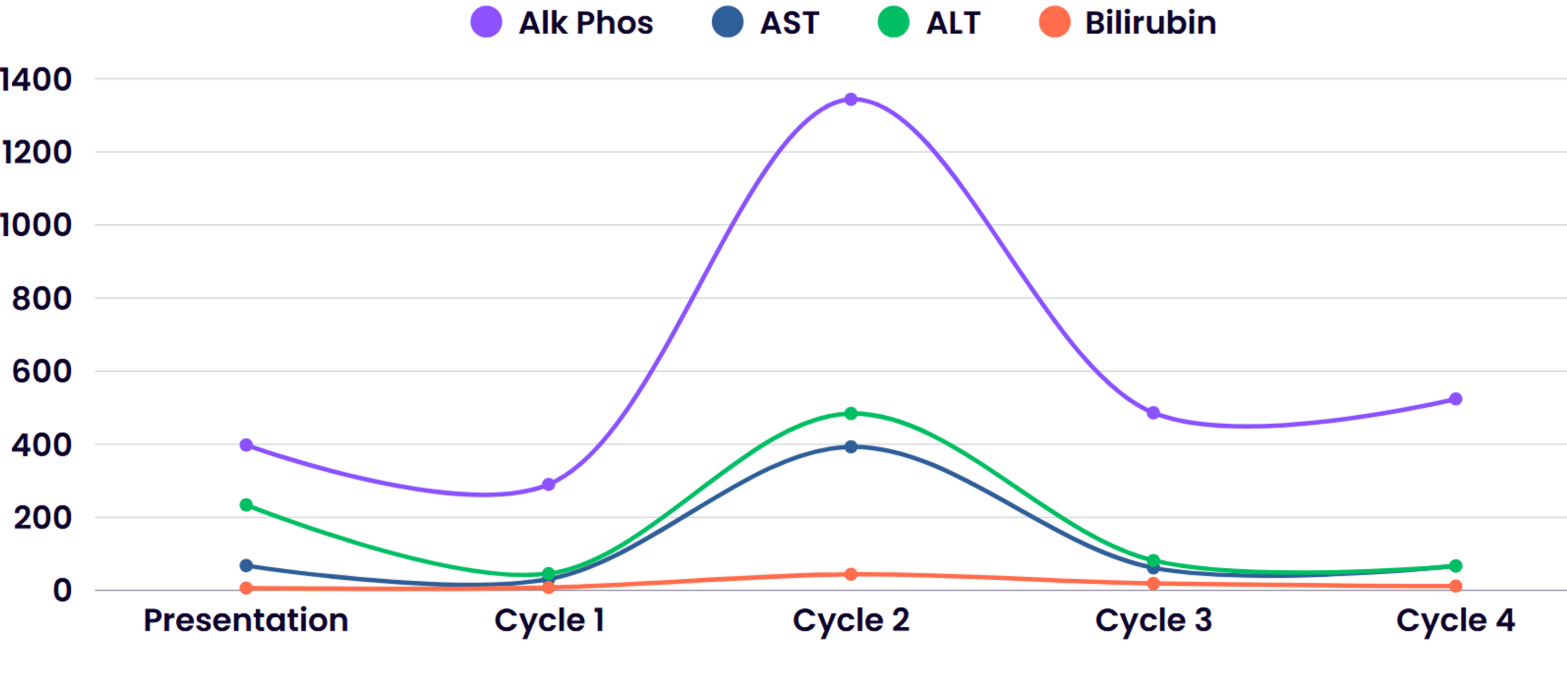
Trend of alkaline phosphatase, AST, ALT, and bilirubin throughout the patient’s presentation Cycle indicates when the patient received chemotherapy. Cycle 1: brentuximab, cyclophosphamide, dexamethasone; Cycle 2: cyclophosphamide, dexamethasone; Cycle 3 and Cycle 4: brentuximab, vinblastine, dacarbazine ALT: alanine transaminase; AST: aspartate aminotransferase

Figure 8 demonstrates the overall trend for the patient’s liver tests throughout his presentation.

The patient was not considered an orthotopic liver transplant candidate, given his active lymphoma and lack of adequate family or social support for post-transplant care. Transplant assessment remains on hold pending completion of Hodgkin's lymphoma treatment, and issues related to care planning, such as securing appropriate caregiver support, are resolved.

## Discussion

The association between VBDS and HL was first described in 1993 by Hubscher et al. [3] and, since then, has been described in a number of published cases. We compiled a list of all published case reports through a search of the PubMed database, Science Direct, and Google Scholar, including both English and non-English cases, which is summarized in Table 2.

**Table 2:** Case report search of VBDS spanning from 1962 to 2025 with descriptions of liver biopsy findings, HL treatment received, and patient outcomes Source: [4-48] HL: Hodgkin’s lymphoma; VBDS: Vanishing bile duct syndrome; IC: Idiopathic cholestasis

Author	Year	Liver biopsy	HL treatment (chemotherapy and/or radiation)	Outcome/cause of death
Hasan F [4]	2024	VBDS	Yes	Unknown
Patodiya B [5]	2023	Lobular inflammation	Yes	Remission
Nano O [6]	2023	VBDS	Yes	Remission
Shokri F [7]	2023	VBDS	Yes	Death/hepatic and renal failure
Keramidas V [8]	2021	VBDS	Yes	Remission
Papakonstantinou I [9]	2021	IC	Yes	Remission
Ousley RJ [10]	2021	VBDS	Yes	Remission
Greca RD [11]	2020	VBDS	Yes	Unknown
Bakhit M [12]	2017	VBDS	Yes	Unknown
Rota Scalabrini D [13]	2014	VBDS	Yes	Remission
Nader K [14]	2013	VBDS	Yes	Death/hepatic failure and sepsis
Aleem A [15]	2013	VBDS	Yes	Death/hepatic failure
Wong KM [16]	2013	VBDS	Yes	Remission
Umit H [17]	2009	VBDS	Unknown	Unknown
Pass AK [18]	2008	VBDS	Yes	Remission/awaiting liver transplant
Pass AK [18]	2008	VBDS	Yes	Death/aspiration
Leeuwenburgh I [19]	2008	VBDS	Yes	Remission
DeBenedet AT [20]	2008	VBDS	Yes	Death/unknown
Ballonoff A [21]	2007	VBDS	Yes	Remission
Barta SK [22]	2006	IC	Yes	Remission
Schmitt A [23]	2006	VBDS	No	Death/sepsis
Han WS [24]	2005	VBDS	Unknown	Recurrent HL
Cordoba Iturriagagaitia A [25]	2005	VBDS	Unknown	Remission
Guliter S [26]	2004	VBDS	Yes	Death/sepsis
Liangpunsakul S [27]	2002	Cholestatic hepatitis	Yes	Remission
Komurcu S [28]	2002	VBDS	Yes	Death/hepatic failure
Ripoll C [29]	2002	VBDS	Yes	Death/hepatic failure
Ripoll C [29]	2002	VBDS	Yes	Remission
Ozkan A [30]	2001	VBDS	Yes	Death/hepatic failure
Allory Y [31]	2000	VBDS	Unknown	Unknown
Rossini MS [32]	2000	VBDS	Yes	Death/hepatic failure
Yusuf MA [33]	2000	VBDS	Yes	Death/hepatic failure
Dourakis SP [34]	1999	Hepatocellular necrosis	Yes	Death/hepatic failure
Yalcin S [35]	1999	IC	No	Death/sepsis
Yalcin S [35]	1999	IC	Yes	Remission
De Medeiros BC [36]	1998	VBDS	Yes	Death/hepatic failure
De Medeiros BC [36]	1998	VBDS	Yes	Remission
Crosbie OM [37]	1997	VBDS	Yes	Remission
Gottrand F [38]	1997	VBDS	No	Death/hepatic failure
Warner AS [39]	1994	IC	Yes	Remission
Jansen PLM [40]	1994	IC	Yes	Death/variceal hemorrhage
Hubscher SG [3]	1993	VBDS	Yes	Death/pneumonia
Hubscher SG [3]	1993	VBDS	Yes	Death/unknown
Hubscher SG [3]	1993	VBDS	Yes	Death/sepsis
Birrer MJ [41]	1987	IC	Yes	Death/sepsis
Lieberman DA [42]	1986	IC	No	Death/respiratory arrest
Trewby PN [43]	1979	IC	Yes	Remission
Trewby PN [43]	1979	Mild portal hepatitis	No	Death
Trewby PN [43]	1979	Lymphoma infiltration	Yes	Death
Trewby PN [43]	1979	Lymphoma infiltration	No	Death
Trewby PN [43]	1979	Mixed inflammatory and atypical histiocytes	Yes	Remission
Trewby PN [43]	1979	IC	Yes	Death/hepatic failure
Piken EP [44]	1979	IC	Yes	Death/unknown
Perera DR [45]	1974	IC	Yes	Death/hepatic failure
Perera DR [45]	1974	IC	Yes	Remission
Perera DR [45]	1974	IC	Yes	Remission
Groth C [46]	1972	IC	Yes	Death/hepatic failure
Juniper K [47]	1963	IC	Yes	Death/hepatic failure
Bouroncle B [48]	1962	IC	Yes	Death/hepatic failure
Bouroncle B [48]	1962	IC	Yes	Death/hepatic failure

Among the 60 case reports listed above, remission was achieved in 36.67% of cases (n=22), not including those patients whose outcomes were unknown. Although liver function test abnormalities and hepatic involvement with HL can be seen in up to 50% of patients due to hemolysis, biliary obstruction from lymph node enlargement, and viral illness such as CMV, VBDS specifically is a rare manifestation of HL patients and overall can be a marker of poor prognosis [12]. Differentiating VBDS from HL involvement of the liver relies on liver biopsy showing histologic evidence of more than 50% interlobular bile duct loss in a specimen that includes at least 10 portal tracts [1]. In severe instances, a biopsy may show a total lack of bile ducts with complete ductopenia, having "burnt-out" portal tracts that are devoid of bile ducts [1]. Diagnosing VBDS can therefore be difficult due to its nonspecific symptoms and the necessity of a liver biopsy to confirm the absence of bile ducts.

The pathophysiology is complex and postulated to be multifactorial, involving autoimmune, inflammatory, and regenerative factors all playing a role in the destruction of bile ducts, though the precise mechanism is unclear [1]. The onset of VBDS is believed to be linked to the heightened apoptosis of biliary epithelial cells, which occurs in response to triggers such as death receptor activation, autoimmune disease, ischemia, oxidative stress, toxins, and viral pathogens. Some theories include cell-mediated immunologic reactions leading to ductopenia, auto-apoptosis of biliary epithelium, or cytokines released by lymphoma cells [1].

VBDS can occur as both an irreversible and a transient condition. While some cases progress to fibrosis, cirrhosis, and liver failure, others may improve over months to years. Liver transplantation is reserved for advanced or refractory cases when the condition leads to significant ductopenia, biliary cirrhosis, and liver failure. However, it is important to consider treatment of the underlying cause and the possibility of biliary regeneration [49-52]. Broadly speaking, VBDS of any origin can be considered for treatment with corticosteroids and ursodeoxycholic acid (UDCA) that may slow inflammation and stimulate bile secretion, preventing apoptosis of cholangiocytes. UDCA utilizes multiple strategies to defend cholangiocytes from the damaging impact of bile acids and to boost the function of impaired hepatobiliary secretion [53-55].

From an oncology perspective, treatment of HL-related VBDS is controversial due to the selection of agents, timing of initiation, and questions regarding liver transplantation referral and candidacy. It overall makes treating an otherwise curable malignancy quite challenging. This is because the standard-of-care treatment for HL is with the first-line regimen known as ABVD: doxorubicin, bleomycin, vinblastine, and dacarbazine. However, doxorubicin and vinblastine are both hepatically metabolized. Doxorubicin is contraindicated if bilirubin is higher than 5.0 mg/dL, and vinblastine is contraindicated if the AST/ALT are greater than three times the upper limit of normal. There is no consensus on first-line therapy in this setting. Whether the modified ABVD regimen should be given with dose adjustment, or if the second-line regimen GDP (gemcitabine 1,600 mg (1,000 mg/m^2^ IV infusion), dexamethasone 40 mg (once daily orally), and carboplatin 600 mg infusion (target AUC of 4-6 mg/mL/min)) should be used is unclear and provider-dependent [13]. Scalabrini et al. reported that only 12% of patients with VBDS who received an upfront reduced ABVD regimen had improved cholestasis and liver function impairment compared to 51% of patients receiving the full dose [13] and therefore advocate for normal dosing. However, it is important to note that these specific patients initially responded well clinically to high doses of UDCA and prednisone prior to receiving ABVD. Conventionally, most other clinicians will attempt modified or alternative regimens to treat the underlying HL until the hyperbilirubinemia normalizes enough to allow ABVD treatment, such as single-agent chemotherapy that is not hepatically metabolized or dose-reduction [6]. Literature review also shows favorable outcomes with the use of nivolumab (a programmed cell death protein or PD-1 receptor inhibitor that prevents T-cell silencing) and brentuximab (an antibody-drug conjugate that binds to cluster of differentiation or CD-30 antigen on cancer cells that disrupts microtubules and leads to cell apoptosis) [5,11,56]. For example, Gupta et al. reported a favorable outcome in patients first given single-agent brentuximab as bridging therapy [57]. Liver transplant was discussed in some of the reviewed case reports as last-line therapy for irreversible cirrhosis; however, active HL disease often precludes these patients from being on the transplant list.

Regarding our patient, he was started on chemotherapy with cyclophosphamide, dexamethasone, and brentuximab vedotin, a combination therapy modified to minimize hepatotoxicity [58] and the potential toxicities of impaired hepatic drug clearance. His labs showed stable liver tests after the first cycle of treatment. Though his liver tests continued to rise thereafter, at the time of writing this article, the patient has completed four cycles of treatment, and his liver tests are improving. He was evaluated by the liver transplant team at a nearby hospital, and a multidisciplinary decision was made to defer transplant until the cHL is completely treated. We posit that liver transplantation should be considered in cHL patients who achieve complete remission after systemic therapy, given the irreversible nature of bile duct loss.

Generally, active malignancy other than within the confines of specific exceptions for liver transplant (e.g., hepatocellular carcinoma, perihepatic cholangiocarcinoma, other emerging indications) precludes consideration for liver transplant for a two- to five-year period following treatment at many centers. Liver transplantation for VBDS in cHL is controversial and likely rare. To our knowledge, this may be one of the first cases in which an orthotopic liver transplant was being considered as treatment for VBDS in an active cHL patient. While these decisions are complex and need to be made in a multidisciplinary setting with the transplant team and oncology, taking into account potential benefits, risks of relapse/recurrence, and post-transplant immunosuppressive risks, we posit that liver transplantation may be a potential consideration in select cHL patients with VBDS that are not improving who achieve complete remission after systemic therapy given the irreversible nature of bile duct loss.

Although VBDS often portends a poor prognosis in HL patients due to the irreversible nature of bile duct loss leading to hepatic failure and death, recent case reports suggest a possibility of reversal or at least improved survival among patients who respond to cancer treatment. This ranges from complete remission with treatment of lymphoma with either total resolution in lab abnormalities or lingering mild isolated elevation in alkaline phosphatase levels without clinical symptoms [12].

## Conclusions

VBDS is a rare paraneoplastic phenomenon of cHL whose pathophysiology is incompletely understood but thought to be related to lymphoma cytokine release or cell-mediated immunologic reactions causing ductopenia. Rarely, it can be reversible with treatment of cHL, but otherwise can progress to acute liver failure with high mortality rates. It is important to recognize VBDS related to cHL early to initiate treatment with careful consideration of the limiting factors and the potential need for early liver transplant evaluation, if clinically indicated. It is important to acknowledge that, while liver transplantation may be a potential consideration if the liver injury fails to improve in those achieving complete remission, the role of liver transplant in this setting remains limited, is controversial, and needs to be determined in a multidisciplinary transplant setting. Further steps include standardizing the treatment of cHL-related VBDS to optimize survival outcomes.
